# PAD4 mediated histone hypercitrullination induces heterochromatin decondensation and chromatin unfolding to form neutrophil extracellular trap-like structures

**DOI:** 10.3389/fimmu.2012.00307

**Published:** 2012-10-04

**Authors:** Marc Leshner, Shu Wang, Carrie Lewis, Han Zheng, Xiangyun Amy Chen, Lorraine Santy, Yanming Wang

**Affiliations:** Department of Biochemistry and Molecular Biology, Center for Eukaryotic Gene Regulation, Pennsylvania State University, University ParkPA, USA

**Keywords:** pad4, hypercitrullination, neutrophil extracellular traps, chromatin decondensation, heterochromatin protein 1, histone modifications

## Abstract

NETosis, the process wherein neutrophils release highly decondensed chromatin called neutrophil extracellular traps (NETs), has gained much attention as an alternative means of killing bacteria. *In vivo*, NETs are induced by bacteria and pro-inflammatory cytokines. We have reported that peptidylarginine deiminase 4 (PAD4), an enzyme that converts Arg or monomethyl-Arg to citrulline in histones, is essential for NET formation. The areas of extensive chromatin decondensation along the NETs were rich in histone citrullination. Here, upon investigating the effect of global citrullination in cultured cells, we discovered that PAD4 overexpression in osteosarcoma U2OS cells induces extensive chromatin decondensation independent of apoptosis. The highly decondensed chromatin is released to the extracellular space and stained strongly by a histone citrulline-specific antibody. The structure of the decondensed chromatin is reminiscent of NETs but is unique in that it occurs without stimulation of cells with pro-inflammatory cytokines and bacteria. Furthermore, histone citrullination during chromatin decondensation can dissociate heterochromatin protein 1 beta (HP1β) thereby offering a new molecular mechanism for understanding how citrullination regulates chromatin function. Taken together, our study suggests that PAD4 mediated citrullination induces chromatin decondensation, implicating its essential role in NET formation under physiological conditions in neutrophils.

## Introduction

Neutrophils serve as an integral part of the body's innate immune system as they are the first line of defense against invading microbes (Kanthack and Hardy, 1894; Nathan, 2006). Upon release from circulation, a chemotactic gradient guides neutrophils to specific sites of infection. Equipped with an arsenal of antimicrobial proteins, neutrophils are then able to rapidly attack and destroy the pathogens they encounter (Wang et al., 2012). Once a neutrophil recognizes its target, the process of phagocytosis commences. First, actin reorganization under the neutrophil membrane brings the pathogen into a phagosome, where the subunits of the membrane associated nicotinamide adenine dinucleotide phosphate (NADPH) oxidase system would assemble to facilitate formation of reactive oxygen species (ROS). These cytotoxic ROS then attack the pathogen following the formation of the phagolysosome—an intracellular structure where the phagosome fuses with numerous antimicrobial peptide-containing granules and lysosomes. Together, ROS and the antimicrobial peptides effectively destroy the microbe (Klebanoff, 1999). Until recently, phagocytosis has been the most widely accepted method by which neutrophils destroy pathogens.

A novel antimicrobial mechanism, by which neutrophil extracellular traps (NETs) mediate bacterial killing, is now being widely accepted (Brinkmann et al., 2004). Neutrophils are observed to generate extracellular chromatin fibers upon activation with interleukin-8 (IL-8), phorbol myristate acetate (PMA), lipopolysaccharide (LPS) and bacteria (Brinkmann et al., 2004). Following activation, neutrophils undergo distinct morphological alterations leading to ultimate NET formation. First the lobular shape of the neutrophil nucleus is lost followed by nuclear envelope disintegration. Then nuclear, cytoplasmic and granular components mix together followed by a rupture of the cell membrane and the release of intact chromatin into the extracellular space (Fuchs et al., 2007). This extracellular chromatin—NETs—is composed of proteases (i.e., elastase and myeloperoxidase) and histones, which render NETs the antimicrobial ability to restrict pathogens at the site of infection and to ultimately destroy the bacteria they come in contact with (Brinkmann et al., 2004). The significance of NETs as an essential host defense mechanism cannot be underestimated. In chronic granulomatous disease (CGD) patients with impaired NADPH oxidase activity and ROS production, neutrophils have poor antimicrobial activity, which is in part due to the inability to produce NETs (Fuchs et al., 2007; Bianchi et al., 2009). Reversely, gene therapy with the NADPH gene in CGD patients restores NET formation and is a viable treatment for this disease (Bianchi et al., 2009).

The discovery of NETs was a landmark discovery in the field of immunology because it established a novel way by which the body can fight off infections. “Netting” neutrophils comprise a significant division of the host innate defense mechanism as evidenced with the finding of extensive extracellular DNA structures at sites of infection. However, too much NETs are implicated in several diseases, such as deep vein thrombosis (Reayi and Arya, 2005) and multiple sclerosis (Mastronardi et al., 2006). Furthermore, genetic studies linked PAD4 with rheumatoid arthritis (Suzuki et al., 2003) and PAD4 in the synovial fluid of RA patients likely produces citrullinated autoimmune antigens during disease progression (Kinloch et al., 2008). Overall, NETs represent a doubled-edged sword in that these extracellular chromatin structures aid the body to eliminate infections but can also cause diseases. By studying the molecular processes underlying NET formation, there is a strong clinical potential to better understand and find means of regulating the pathological conditions caused by NETs.

NETs are comprised of extensively decondensed chromatin, suggesting that higher-order chromatin is involved in NET formation. In the eukaryotic nucleus, 147 bp of DNA tightly associates with the histone octamer (two of each histones H3, H2B, H2A, and H4) to form a nucleosome—the basic structural unit of chromatin (Richmond and Davey, 2003). The subsequent binding of linker histone H1 further compacts the DNA to form 30 nm chromatin fibers (Horn and Peterson, 2002). It is well recognized that the formation of higher-order chromatin and its structural changes are integral for regulating gene expression (Schalch et al., 2005). A prominent hallmark of NETosis is the rapid decondensation of nuclear chromatin into 15–25 nm chromatin fibers (Brinkmann et al., 2004), which suggests that there is a regulatory mechanism leading to NET formation at the level of chromatin structure regulation. Although the exact mechanisms that control chromatin structure during specific nuclear events remain to be tested, posttranslational histone modifications are known to play a significant role. Acetylation, methylation and phosphorylation of histone proteins regulate chromatin functions, such as transcription as well as chromatin condensation and decondensation (Shilatifard, 2006; Kouzarides, 2007; Li et al., 2007). Heterochromatin binding protein 1 (HP1) is a well-established non-histone protein that binds to modified histones in particular histone H3 Lys9 methylation and regulates chromatin structure. Maintenance of a heterochromatin state relies on the recruitment of HP1 to methylated histone H3 (Verschure et al., 2005). Thus via HP1 binding to specific sites on chromatin, a cell is able to tightly regulate a gene active or inactive state depending on the need for specific cellular functions.

During the search for how neutrophils regulate higher-order chromatin, our lab discovered that peptidylarginine deiminase 4 (also called PAD4 or PADI4) catalyzed histone hypercitrullination mediates chromatin decondensation and is essential for NET formation (Wang et al., 2009; Li et al., 2010). PAD4 is a neutrophil enriched nuclear enzyme that targets histone arginine and mono-methylarginine residues for citrullination in a calcium dependent reaction (Nakashima et al., 2002; Wang et al., 2004). Significantly, extensive citrullination is correlated with chromatin decondensation (Neeli et al., 2008; Wang et al., 2009). Research on PAD4 demonstrates that histone modifications play a substantial role in the change of higher-order chromatin from a condensed to a highly decondensed state during NET formation. Additionally, we have shown that PAD4 is an essential factor for NET-mediated innate immune functions (Li et al., 2010).

In the past decade, ample research has been done to try to dissect the mechanism of NET-mediated bacterial killing to better understand the immune system and how neutrophils respond to infection. Although many mechanisms have been proposed, we hypothesize that the molecular events underlying NET formation involve a global reorganization of chromatin. We postulate that gene regulation plays a significant role in NET formation and that the histone-modifying enzyme PAD4 is one of the main mediators of this immunological process. In the process of preliminary research for PAD4, we unexpectedly observed that mere PAD4 overexpression in osteosarcoma U2OS cells yielded extracellular fibers similar to those observed in NETs. The released chromatin stains strongly by a histone citrulline-specific antibody analogous to what has been observed in the literature upon stimulation of neutrophils with IL-8, PMA, LPS, etc. (Neeli et al., 2008; Wang et al., 2009; Li et al., 2010). Moreover, histone hypercitrullination after PAD4 overexpression excludes HP1β from binding to chromatin. This offers a new mechanism by which PAD4 is able to regulate higher-order chromatin structures to induce NET formation. Our findings reinforce the notion that the regulation of chromatin by histone modifications is essential for the process of NET formation.

## Results

### PAD4 induces extensive chromatin decondensation in non-granulocytic cells

**Figure 1:**
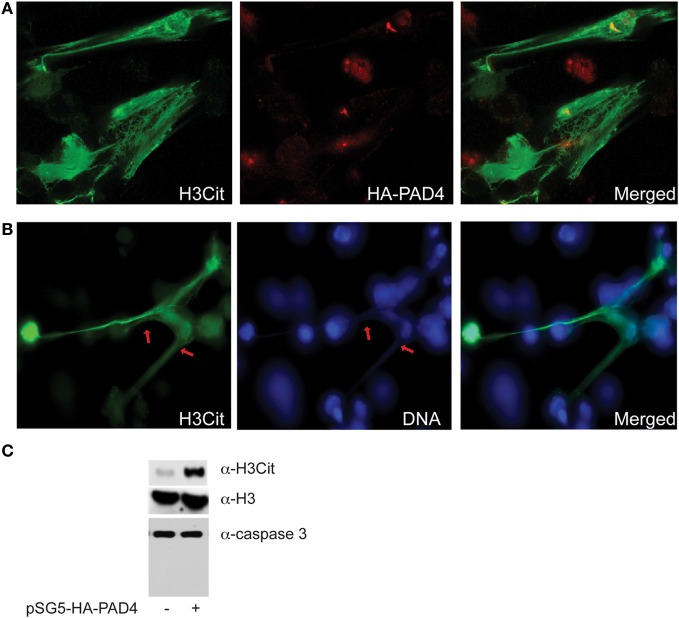
**Dramatic chromatin decondensation and formation of NET-like structures upon forced PAD4 expression. (A)** Immunostaining of U2OS cells with the H3Cit and the HA antibodies after forced HA-PAD4 expression by transient transfection. Note the dramatic global histone H3 hypercitrullination. **(B)** Immunostaining of H3Cit and DNA staining showing the enrichment of H3Cit with the highly decondensed chromatin denoted by red arrows. **(C)** Western blot analyses of the H3Cit levels, and the caspase-3 cleavage in U2OS cells with or without forced HA-PAD4 expression. Histone H3 was probed to ensure equal protein loading.

To elucidate the role of PAD4 in chromatin decondensation, we overexpressed PAD4 in osteosarcoma U2OS cells via transient transfection of a plasmid containing the full-length PAD4 gene. Strikingly, transient transfection of PAD4 for 36 h induced U2OS cells to rupture and release extensive web-like chromatin fibers into the extracellular space (Figure 1). *In vitro*, neutrophils undergo NETosis in the presence of pro-inflammatory cytokines, bacteria, or after calcium ionophore treatment (Brinkmann et al., 2004; Fuchs et al., 2007; Li et al., 2010). However, we observed NET-like structures devoid of any of these stimulants in a non-granulocytic cell line, suggesting that increased PAD4 activity induces the formation of these structures. To assess the similarity between these extracellular fibers and NETs induced *in vitro*, U2OS cells were stained with α-H3Cit, α-HA to localize HA-PAD4 and Hoechst following PAD4 overexpression. We found that H3Cit staining was greatly increased at areas of highly decondensed chromatin (Figures 1A,B). However, PAD4 was not strongly detected there (Figure 1A), suggesting that PAD4 dissociates from the NET-like structure upon rupture of the nuclear and cell membranes. Moreover, the H3Cit antibody stained chromatin was only weakly labeled with the Hoechst reagent, suggesting that the underlying chromatin is highly decondensed. Taken together, these results support that PAD4 overexpression and hypercitrullination of chromatin led to the formation of highly decondensed chromatin structures reminiscent of NETs.

A previous study hinted that PAD4 overexpression induces apoptosis due to release of cytochrome *c* and activation of caspases (Liu et al., 2006). To further analyze the nature of the cell death elicited by PAD4 overexpression, Western blot analyses were performed using U2OS cell lysate with or without PAD4 overexpression. An increase in H3Cit but no cleavage of caspase-3 was detected in cells over-expressing PAD4 as compared to the control cells (Figure 1C). Given that caspase-3 activation was not detected and the released chromatin remained intact after PAD4 overexpression, we favor that PAD4 overexpression induced the NETosis type of cell death instead of apoptosis under above experimental conditions in the U2OS cells.

### Chromatin released from U2OS cells is similar to NETs

**Figure 2:**
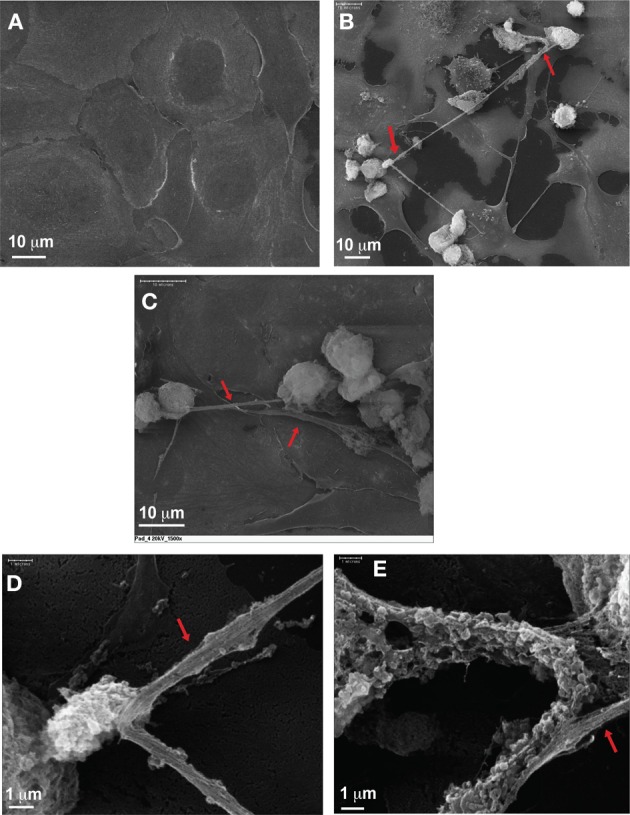
**Scanning electron microscope analyses of extracellular chromatin fibers. (A)** U2OS cells without forced HA-PAD4 expression. **(B–E)** U2OS cells with forced HA-PAD4 expression, showing the decondensed chromatin fibers (denoted by red arrows). Also noticeable is the membrane vesicles attached to the chromatin fibers in **(E)**.

It was apparent that the “NET-like” structures following transient transfection of PAD4 in U2OS cells correlated, at least from an immunohistochemical standpoint, with previous studies examining NETs derived from neutrophils. However, it is important to note that neutrophil released chromatin assumes a distinct morphology and is not surrounded by membrane (Brinkmann et al., 2004). To further evaluate the chromatin structures formed by PAD4 overexpressing U2OS cells, scanning electron microscopy (SEM) was performed. U2OS cells that did not overexpress PAD4 were flat, attached firmly to the cover slip with visible nuclei and no extracellular fibers (Figure 2A). However, after PAD4 overexpression, many cells became round and lost attachment to the substratum (Figures 2B,C). Upon closer examination, transfected cells made prominent extracellular fiber structures analogous to NETs (Figure 2, **denoted by red arrows**). These structures assumed thin and thick stretches that literally extruded from the cells and in some instances this fibril network encompassed the entire cell it originated from (Figure 2D). Furthermore, high magnification images showed vesicular membrane structures interspersed within the larger chromatin stretches (Figure 2E). These results offer supporting evidence that PAD4 overexpressing cells release “NET-like” structures into the extracellular space.

### PAD4 mediated chromatin decondensation is dependent on the activity of PAD4

**Figure 3:**
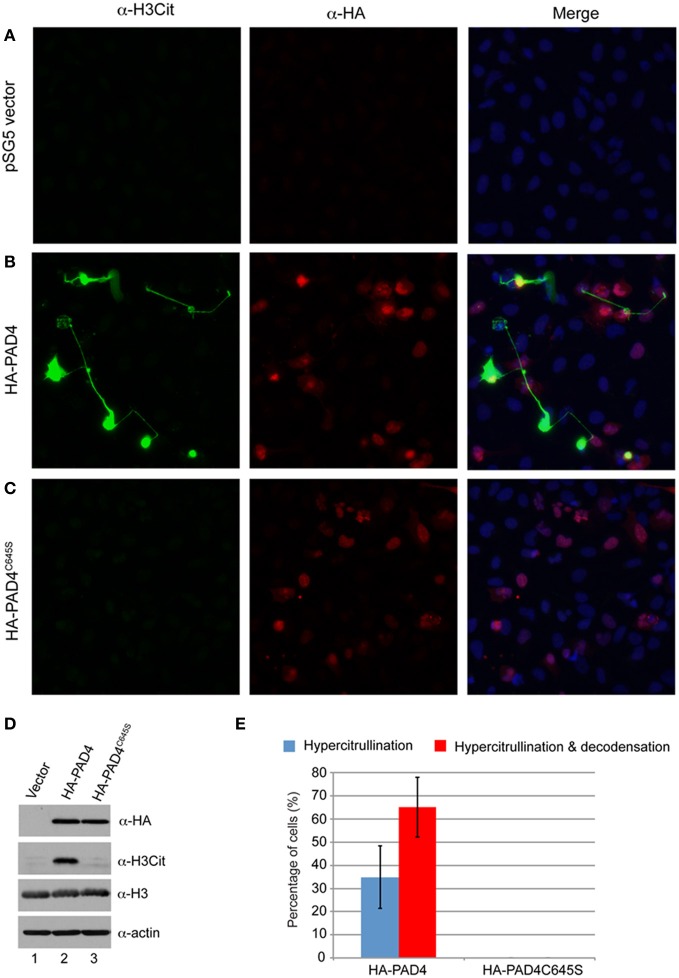
**PAD4 activity is important for the induction of NET-like structures. (A–C)** Fluorescent microscope analyses of histone H3Cit and chromatin morphology in U2OS cells transfected with the pSG5 vector, the pSG5-HA-PAD4 plasmid, or the pSG5-HA-PAD4^C645S^ plasmid. **(D)** Western blot analyses of HA-fusion protein expression, histone H3Cit levels in U2OS cells after transient transfection. Histone H3 and actin were probed to ensure equal protein loading. **(E)** The number of H3Cit positive cells without obvious chromatin decondensation (hypercitrullination) or H3Cit positive cells with obvious chromatin decondensation (hypercitrullination and decondensation) were numerated as a percentages of cells that are H3Cit positive in U2OS cells transfected with the pSG5-HA-PAD4 plasmid or the pSG5-HA-PAD4^C645S^ plasmid.

If mere PAD4 protein elevation and histone citrullination can induce chromatin decondensation, it is expected that the relative enzymatic activity of PAD4 is essential for this process. To test this idea, we analyzed the NET-induction ability of a plasmid expressing an enzymatically inactive PAD4 mutant—HA-PAD4^C645S^—in U2OS cells. As controls, we found that the pSG5 plasmid vector alone did not induce NET-like structures (Figure 3A), while pSG5-HA-PAD4 plasmid did (Figure 3B). In contrast, after transient transfection of the pSG5-HA-PAD4^C645S^ plasmid, NET-like structures were not detected, suggesting that the activity of PAD4 is required for the NET-like structure induction (Figure 3C). The equal amount expression of the HA-PAD4 protein or the HA-PAD4^C645S^ mutant protein was detected by Western blot (Figure 3D). Consistent with the immunostaining experiments, histone H3 citrullination was detected by the H3Cit antibody only in cells with the forced expression of the HA-PAD4 protein (Figure 3D). The amount of histone H3 and actin was also monitored to ensure equal protein loading (Figure 3D, two bottom panels). The number of cells that are positive for H3 hypercitrullination staining or double positive for both histone H3 hypercitrullination and chromatin decondensation from independent fields in immunostaining experiments was tabulated as percentage of total H3Cit positive cells and displayed in a bar graph (Figure 3E). This quantification indicated that cells positive for only citrullination or double positive for both citrullination and chromatin decondensation were detected after the expression of HA-PAD4 but not after the expression of HA-PAD4^C645S^ (Figure 3E). Taken together, these results support the notion that the PAD4 activity is crucial for extensive chromatin decondensation.

### PAD4 mediated chromatin decondensation is calcium dependent

**Figure 4:**
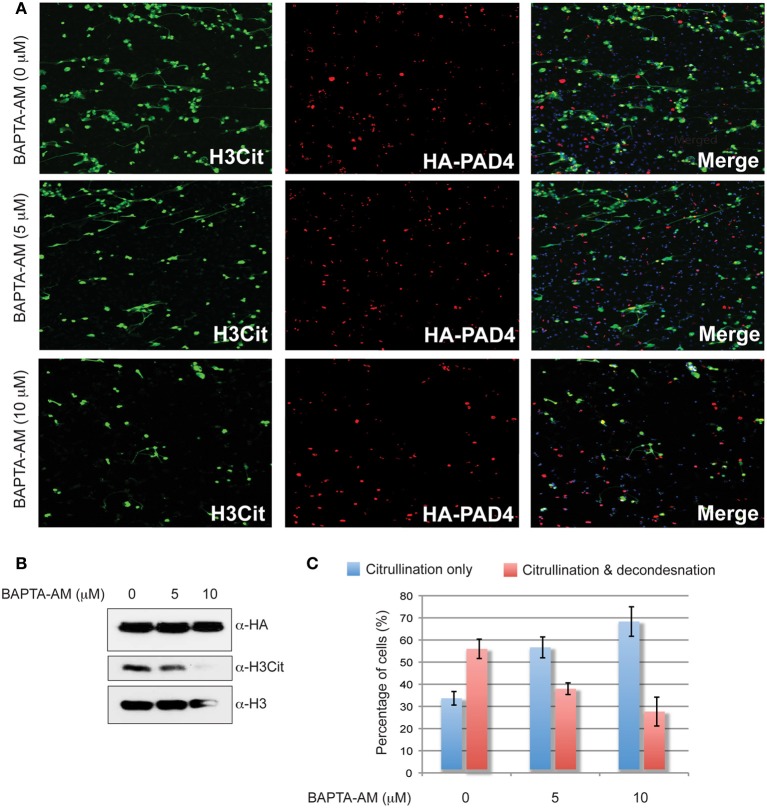
**The calcium chelator BAPTA-AM attenuates histone hypercitrullination and chromatin decondensation induced by forced HA-PAD4 expression. (A)** Fluorescent microscope analyses of histone H3Cit levels in U2OS cells transfected with the pSG5-HA-PAD4 plasmid and then treated with BAPTA-AM at 0, 5, and 10 μM concentrations. **(B)** Western blot analyses of histone H3Cit levels in U2OS cells transfected with the pSG5-HA-PAD4 plasmid then treated with BAPTA-AM. Histone H3 blot was performed to show the amount of histone H3 in each sample. HA western blot was performed to monitor the HA-PAD4 expression. **(C)** The number of H3Cit positive cells without obvious chromatin decondensation (citrullination only) or H3Cit positive cells with obvious chromatin decondensation (citrullination and decondensation) were numerated as a percentages of cells that are H3Cit positive in U2OS cells transfected with the pSG5-HA-PAD4 plasmid and then treated with BAPTA-AM at different concentrations.

Using the U2OS cell system for analyzing extensive chromatin decondensation makes it possible to dissect the molecular processes leading to this event. Literature has shown that *in vivo* citrullination was induced when cells were treated with calcium ionophore (Takahara and Sugawara, 1986; Vossenaar et al., 2003). Interestingly, after PAD4 overexpression in U2OS cells, we observed abundant citrullination without calcium ionophore treatment, raising a question if calcium elevation is required for histone citrullination under the condition of PAD4 overexpression. To test this idea, we employed an intracellular calcium chelator 1,2-Bis(2-aminopheoxy)ethane-N,N,N',N'-tetraacetic acid tetrakis (acetoxymethyl ester), better known as BAPTA-AM (Takahashi et al., 1999), at non-toxic concentrations. HA-PAD4 was first expressed in U2OS by transient transfection. Cells were then treated with 0, 5, and 10 μM BAPTA-AM, followed by fixation and immunostaining with α-HA-PAD4 and α-H3Cit antibodies as well as Hoechst. A similar experiment was performed in parallel to prepare protein samples for Western blot analyses. After treatment with an increasing amount of BAPTA-AM, an apparent decrease in the extent of cells undergoing histone citrullination and chromatin decondensation was detected (Figure 4A). Moreover, western blot analyses detected equal amount of PAD4 expression but reduced levels of H3Cit after treatment with an increasing amount of the calcium chelator (Figure 4B), suggesting that calcium is important for histone citrullination. The number of cells that are positive staining of H3 citrullination or that are double positive for both H3 citrullination and chromatin decondensation in three independent experiments was tabulated as a percentage of H3Cit positive cells and displayed in a bar graph (Figure 4C). We found that at increasing concentrations of BAPTA-AM, the percentage of cells that were solely H3Cit positive increased while the percentage of cells that were H3Cit and decondensation double positive decreased (Figure 4C). These results reveal that U2OS cells must achieve sufficient intracellular calcium concentrations to induce PAD4 mediated histone citrullination and chromatin decondensation.

### Histone citrullination induces HP1β dissociation and heterochromatin decondensation

**Figure 5:**
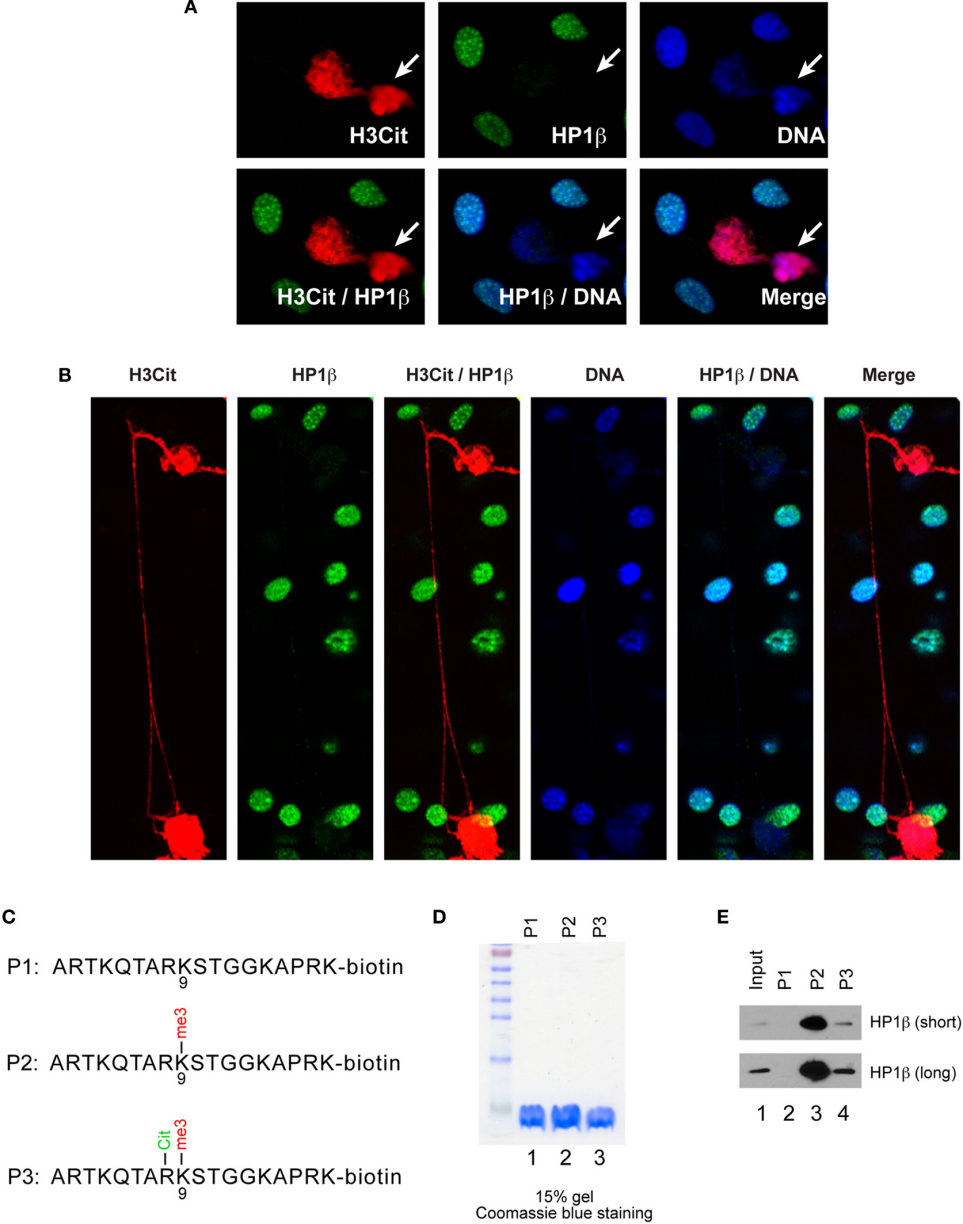
**Forced HA-PAD4 expression induced heterochromatin decondensation and HP1β dissociation in NIH 3T3 cells. (A)** Immunostaining assays of H3Cit and HP1β in NIH 3T3 cells after forced HA-PAD4 expression. Arrows denote a cell with an increase in H3Cit, a loss of HP1β and the organization of distinct heterochromatic loci, a distinct feature of these cells. **(B)** Immunostaining with H3Cit and HP1 β in NIH 3T3 cells with forced HA-PAD4 expression. Note the extreme chromatin decondensation in the extracellular space. **(C)** The sequence of the biotin conjugated H3 N-terminal peptides with no modification, K9me3, or Cit8K9me3 modifications. **(D)** Coomassie blue staining to show the amount of each peptide. **(E)** Peptide pull-down experiments to analyze the binding of HP1β to H3 unmod, K9me3, and Cit8K9me3 peptides. Top panel shows a short exposure and bottom panel shows a long exposure time in Western blot assays.

That PAD4 overexpression induces chromatin decondensation underscores the significance of chromatin modifiers and posttranslational modifications in NET formation. However, the exact role of citrullination at histone arginine residues to induce chromatin decondensation is still unclear. Several possible mechanisms can be envisioned. For example, global hypercitrullination of histones neutralizes the net positive charge of chromatin and as a result there is a loss of electrostatic interaction between DNA and histones leading to chromatin decondensation. Alternatively, citrullination at arginine residues serve as an epigenetic mark to recruit additional chromatin modifiers that “unravel” chromatin. Moreover, it is possible that histone citrullination prevents the binding of known chromatin condensation factors. An understanding of how the higher-order chromatin state is altered leading to chromatin decondensation will provide a molecular mechanism by which NET formation occurs. The heterochromatin protein 1 (HP1) family members are chromatin regulators that bind to H3 Lys9 methylated residues to regulate heterochromatin formation and function. Overexpression of HP1β is sufficient to induce local chromatin condensation. More significantly, the binding of HP1β to chromatin is regulated by posttranslational histone modifications such as methylation and phosphorylation (Fischle et al., 2005; Verschure et al., 2005). Since a preferred target of PAD4, the H3 Arg8 residue, is adjacent to H3 Lys9, we hypothesized that citrullination of Arg8 affects the binding of HP1β to methylated histone H3 and thereby perturbing the function of heterochromatin during NET formation. To test this hypothesis, we employed NIH 3T3 cells, a mouse fibroblast cell line well known for the formation of discrete regions of heterochromatin within the nucleus, with foci of condensed chromatin stained by the HP1β mouse monoclonal antibody (Figure 5A). We found that overexpression of HA-PAD4 in NIH 3T3 cells induced histone H3 citrullination detected by the H3Cit antibody staining (Figure 5A) as well as extensive chromatin decondensation showing strong H3Cit antibody staining (Figure 5B). This experiment was crucial for illustrating that PAD4 mediated chromatin decondensation after PAD4 overexpression can occur in other cells lineages besides U2OS cells, i.e., PAD4 mediated NET-like structure formation is not cell type specific. Interestingly, cells positive for H3Cit or double positive for H3Cit and chromatin decondensation did not stain with the HP1β antibody (Figures 5A,B). Furthermore, cells stained strongly with the H3Cit antibody lost the foci of dense heterochromatin (Figure 5A, denoted by arrows). These results suggest that mere PAD4 overexpression can decondense euchromatin and heterochromatin. It has recently been reported that a synthetic H3Arg8Cit peptide can prevent the binding of H3K9me2/3 to HP1β (Bock et al., 2011). The fact that HP1β is lost in H3Cit positive cells promoted us to assess whether H3 Arg8 citrullination adjacent to the methylated Lys9 residue inhibits the binding of HP1β. Toward this end, three H3 N-terminal peptides were synthesized (illustrated in Figure 5C). Peptide 1 (P1, H3 residues 1–18) was unmodified with C-terminal biotin conjugation. Peptide 2 (P2) is the same as P1 but contains K9me3 modification, while peptide 3 (P3) contains Cit8 and K9me3 dual modification. The amount of the three peptides was analyzed in a 15% SDS-PAGE gel followed by Coomassie blue staining (Figure 5D). In peptide pull down experiments, H3K9me3 peptide but not the H3 unmodified peptide was able to retain HP1β (Figure 5E). In contrast, the efficacy of HP1β interaction with H3K9me3 was significantly decreased by the Cit8 modification at the neighboring Arg8 residue (Figure 5E), suggesting that histone H3 citrullination in particular H3Cit8 modification can regulate the binding of HP1β to chromatin thereby the organization of high order chromatin structure.

This observation is consistent with the idea that PAD4 is released from dying and NET-forming neutrophils in the joint of RA patients during disease progression to produce citrullinated autoimmune antigens.

## Discussion

In this study, we showed that (1) PAD4 overexpression causes extensive chromatin decondensation in non-granulocytic cells in a manner similar to NETs, (2) the process of extensive chromatin decondensation is dependent upon the enzymatic activity of PAD4 and sufficient intracellular calcium concentrations, and (3) citrullination prevents the binding of HP1β to chromatin. This study supports the essential role of PAD4 in inducing NET formation and provides a potential model system for assessing the biological role of PAD4 in this process.

PAD4 is responsible for histone “hypercitrullination” found along NETs as well as required for bacterial killing mediated by these chromatin “webs” (Neeli et al., 2008; Wang et al., 2009; Li et al., 2010). These findings suggest a role for histones and their epigenetic modifications in a physiological process that was not fully recognized. Here we show that PAD4 overexpression in non-granulocytic cells devoid of pro-inflammatory cytokines or calcium ionophore treatment triggers extensive chromatin decondensation. The decondensed chromatin stained positive for histone citrullination, similar to NETs and upon further analysis occurred without caspase-3 cleavage, a prominent mark of cell death via apoptosis. Other work has also shown that NET formation results in neutrophil death in a manner independent from apoptosis because NET-forming cells do not display “eat-me signals” such as phosphatidyl-serine and no caspase activity is detectable (Remijsen et al., 2011). Our results therefore illustrate that PAD4 overexpression triggers extensive chromatin decondensation biochemically and morphologically analogous to NETs in a manner independent of apoptosis in U2OS cells.

As previously stated, immunofluorescence studies following transfections experiments validated that PAD4 is crucial for mediating chromatin decondensation. However, to further solidify our findings, it was necessary to closely examine the “NET-like” structures formed by PAD4 overexpressing cells. Some of the primary literature examining NETs provides electron microscopy studies illustrating that neutrophils undergoing NETosis form extensive membrane protrusions and the released DNA forms a meshwork of fibers (Brinkmann et al., 2004). Similarly, our SEM analysis revealed that PAD4 overexpressing cells emanated both long and dense stretches of chromatin. The denser areas of fibril matrix were also marked by vesicular structures probably the result of the chromatin interaction with the cell membrane before extracellular release. Taken together, the fluorescent and EM microscopic analyses showed the great similarity between U2OS derived extracellular chromatin fibers and NETs.

Although PAD4 is a crucial mediator of NETosis (Li et al., 2010), how PAD4 fits into the molecular mechanism for inducing NET formation is poorly understood. NETosis induction requires pre-treated with calcium ionophore to promote global activation of PAD4 in primary neutrophils or HL-60 granulocytes (Takahara and Sugawara, 1986; Wang et al., 2009). Inhibition of PAD4 with Cl-amidine followed by calcium ionophore treatment of differentiated HL-60 cells results in a significant reduction of histone citrulline positive decondensed chromatin (Wang et al., 2009). Intriguingly, our experiments showed that chromatin decondensation could be induced in cells without treatment with calcium ionophore. Moreover, chelating intracellular calcium with BAPTA-AM in PAD4 overexpressing cells results in a marked decrease in decondensed chromatin with double positive H3Cit staining and chromatin decondensation. Our results thus highlight a possible calcium regulated mechanism leading to PAD4 mediated chromatin decondensation. Under physiological conditions, calcium is sequestered in subcellular organelles to prevent aberrant signaling cascades. From the perspective of PAD4, the intracellular calcium concentration of 10^−8^ – 10^−6^ molar is required for robust PAD4 activation (Takahara and Sugawara, 1986). Our work shows blocking calcium signaling by a chelator can inhibit PAD4 overexpression-mediated NET formation. As calcium triggers multiple signaling events, the cellular pathways leading to a full activation of PAD4 remains unknown. The notion that the activity of PAD4 is needed for chromatin decondensation is further supported from our study employing the inactive mutant PAD4^C645S^.

To date, the mechanism of NETosis has been tackled at the macroscopic level via visualization of live neutrophils stimulated for NET formation. From these studies, we know that prior to chromatin release there is a loss of nuclear integrity, mixing of chromatin with cellular granules and finally, disintegration of the nuclear membrane (Medina, 2009). Additionally, from a signaling standpoint, CXCR2 and Src family kinases seem to mediate NET formation (Marcos et al., 2010). However, there seems to be a lack of emphasis on gene regulation and alteration of higher order chromatin structure as an underlying mechanism leading to NET formation. Overall our work has demonstrated that PAD4 is an chromatin modifier that alters the structure of chromatin via catalyzing citrullination at Arg residues on histones (Wang et al., 2009), and is required for NET-mediated bacterial killing (Li et al., 2010) and PAD4 triggers chromatin decondensation in non-granulocytic cells (this study). Therefore the mechanism of NET formation, i.e., the extensive “unraveling of nuclear chromatin,” is tightly linked to PAD4 and histone hypercitrullination.

**Figure 6:**
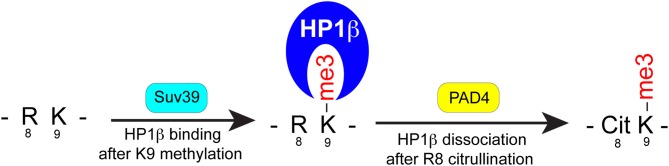
**Working model for the effects of H3Arg8 (R8) citrullination on the binding of HP1β to H3K9me3.** (1) Upon methylation of H3K9 by the methyltransferase (e.g., Suv39), HP1β recognizes this modification and is recruited to a chromatin region to regulate heterochromatin structure and gene repression. (2) Reversely, PAD4-catalyzed citrullination of H3R8 produce a dual histone H3 modification—H3Cit8K9me3—to dissociate HP1 and mediate heterochromatin decondensation during NET formation.

One of the best-characterized proteins associated with chromatin condensation is HP1 (Eissenberg and Elgin, 2000). HP1 preferentially binds to histone H3K9me3 residues allowing for nucleation of chromatin into a highly condensed state (Verschure et al., 2005). Our studies support that citrullination may antagonize the binding of HP1β to chromatin allowing the cell to decondense heterochromatin during NET formation. It has recently been shown in peptide array assays that H3Cit8 will inhibit the binding of HP1β to the H3K9me3 (Bock et al., 2011). HP1β is negatively regulated by other post-translation modification, such as by the phosphorylation of serine 10 adjacent to methyl-lysine 9 (Fischle et al., 2005). The fact that citrullination affects the binding of HP1β to chromatin suggests another mechanism for PAD4 mediated chromatin decondensation during the process of NET formation. Based upon our findings we can derive a model for PAD4 mediated chromatin decondensation as occurs in neutrophils undergoing NETosis (Figure 6). In non-stimulated neutrophils, nuclear heterochromatin is regulated by HP1β bound to H3K9me2/3. Upon stimulation, PAD4 is activated and globally citrullinates Arg8 residues adjacent to the K9me2 or K9me3 residues. The shift in equilibrium to chromatin in a more citrullinated state would prevent HP1 from binding to chromatin thus promoting chromatin decondensation.

NET formation is a truly unique but also effective immune response that illustrates the importance of histone modifications at the level of higher-order chromatin in inducing a physiological process. PAD4 plays a prominent role in inducing the extensive chromatin decondensation that occurs in NET producing cells. With a better understanding of how NETs form and the role PAD4 plays in the process, it is possible to find ways of controlling NET-associated diseases such as systematic lupus erythematousus (SLE), deep venous thrombosis (DVT), preeclampsia, etc. As we have recently shown that the newly developed PAD4 inhibitors could serve as putative cancer therapeutics (Wang et al., 2012), inhibiting PAD4 could offer a new strategy for treatment of NET associated ailments.

## Methods

### Cell culture and transient transfection

U2OS and NIH 3T3 cells were cultured in DMEM medium supplemented with 10% FBS and 1% Penicillin-Streptomycin in a 5% CO_2_ incubator at 37°C. To start the transient transfection, 2–3 × 10^5^ U2OS or NIH 3T3 cells were plated in a 6-well plate. Replace the medium to fresh medium without antibiotics early in the morning on the day of transfection. When cells reached ~70–90% confluence, 4 μg of DNA (pSG5-HA-PAD4 or pSG5-HA-PAD4^C645S^) was diluted with 250 μl OPTI-MEM and 10 μl of Lipofectamine 2000 (Invitrogen) was combined with 240 μ l OPTI-MEM and incubated for 5 min at RT. The DNA/OPTI-MEM and Lipofectamine 2000/OPTI-MEM was combined and incubated for 20 min at RT. Five-hundred microliters of plasmid/lipofectamine complex was added to the six-well plate and then placed in a 5% CO_2_, 37°C incubator. After 12 h, the transfection medium was replaced with fresh complete medium.

### Immunostaining and fluorescent microscopy

Immunostaining was performed using a previously established protocol. After fixation of samples with 3.7% paraformaldehyde in PBS supplemented with 1% Triton X-100 and 2% NP-40, cells were washed with PBST three times 10 min each. Following the third wash, cells were blocked in 2% BSA in PBST for at least 2 h at RT. Primary antibodies were diluted in PBST supplemented with 2% BSA and 5% normal goat serum as follows: α-HA (Sigma, H9658, mouse mAb, 1:200 dilution), α-H3Cit (Abcam, Ab5103, rabbit pAb, 1:200 dilution), and α-HP1β (Active motif, 39979, 1:200, dilution). Cellular staining was performed in a humid chamber overnight at 4°C. After application of the primary antibodies, the cells were washed with PBST three times 10 min each. Cells were then stained with the appropriate secondary antibodies conjugated with Cy3 or Alexa488 at a 1:500 dilution in a humid chamber for at least 2 h at RT. After washing three times 10 min each with PBST, cells were stained with 1 μg/ml Hoechst (Sigma, 94403) in PBS for 15 s followed by a final wash with H_2_O. Slides were then mounted and imaged with a fluorescent microscope (Axioscope 40; Carl Zeisss, Inc.). Fluorescent images were captured via an AxioCam MRM camera (Carl Zeiss, Inc.) using the Axiovision AC software (Carl Zeiss, Inc.). Confocal fluorescent images were also captured at the Center for Quantitative Cell Analysis at the Pennsylvania State University. Images were later processed and manipulated using the Adobe Photoshop program or the Image J program as appropriate.

### Protein extraction and western blot

For Western blot analyses, cells were lysed in an appropriate volume of cold IP buffer (10 mM Tris-HCl pH 8.0, 2 mM EDTA, 150 mM NaCl, 0.2% Triton X-100, and 0.2% NP-40) supplemented with protease inhibitors (1 mM PMSF, 1 μg/mL leupeptin, 1 μg/mL aprotinin, and 1 μg/mL pepstatin). Crude extract was then sonicated for ~5 min at 4°C followed by SDS denaturation. The appropriate volume of denatured protein was separated in a 14% SDS-PAGE gel. Proteins were then transferred to nitrocellulose membrane, using a Semi-Dry Transferring system for 1 h. Following Ponceau S staining, the membrane was blocked in 5% fat free milk in TBST for ~30 min at RT to which the following primary antibodies were added: α-H3Cit (AbCam, Ab5103, 1:2000 dilution), α-HA (Sigma, H9658, 1:1000 dilution), α-histone H3 (AbCam, Ab1791, 1:3000 dilution), and α-HP1β (Active motif, 39979, 1:200, dilution). Following overnight incubation at 4°C, membranes were washed 3 times 10 min each in TBST and were then incubated for a minimum of 2 h at 4°C with the proper horseradish peroxidase-conjugated secondary antibody. Signals were detected using the Lumi-Light PLUS Western blotting substrate (Roche Inc.).

### Cellular chromatin decondensation assay and decondensation assay with BAPTA-AM

To assess cellular chromatin decondensation as a result of PAD4 overexpression, U2OS cells were transiently transfected with 4 μg of PSG5-HA-PAD4, a construct that allows detection of HA-PAD4 protein because of the HA epitope. After 12 h of transfection, the medium was removed and replaced with fresh DMEM with antibiotics. At this point, BAPTA-AM (Sigma) was added to 5 and 10 μM final concentrations for treatment. After additional 24 h, the medium was removed and the cells were immunostained with α-HA and α-H3Cit and viewed with fluorescence microscopy to observe increased PAD4 expression and chromatin decondensation.

### Scanning electron microscopy (SEM)

For SEM imaging, U2OS cells after pSG5-HA-PAD4 transfection to induce chromatin decondensation were fixed in 2% glutaraldehyde in 0.1 M pH 7.4 phosphate buffer. Dehydrate is performed by gradual wash of ethanol followed by the CO_2_ critical time point dry. SEM analyses of the morphology of treated or control cells were performed at the Penn State Electron Microscopy Facility.

### Peptide pull-down assays

C-terminal Biotin conjugated H3 peptides (residues 1–18) were synthesized by Peptide 2.0 Inc. About 10 μg of each peptide was incubated with the streptavidin beads (Pierce, 20349) then washed with IP buffer (10 mM Tris-HCl pH 8.0, 2 mM EDTA, 150 mM NaCl, 0.2% Triton X-100, and 0.2% NP-40) freshly supplemented with protease inhibitors. Nuclear extracts were prepared from NIH 3T3 following a previously described protocol (Li et al., 2008). After incubation with the nuclear extracts, peptide-streptavidin beads were washed with IP buffer three times 10 min each. The retained proteins were analyzed by Western blot analyses with the HP1β antibody.

### Conflict of interest statement

The authors declare that the research was conducted in the absence of any commercial or financial relationships that could be construed as a potential conflict of interest.
